# Synergic Effect of Honey with Other Natural Agents in Developing Efficient Wound Dressings

**DOI:** 10.3390/antiox12010034

**Published:** 2022-12-24

**Authors:** Angela Spoială, Cornelia-Ioana Ilie, Denisa Ficai, Anton Ficai, Ecaterina Andronescu

**Affiliations:** 1 Department of Science and Engineering of Oxide Materials and Nanomaterials, Faculty of Chemical Engineering and Biotechnologies, University Politehnica of Bucharest, 1-7 Gh. Polizu Street, 011061 Bucharest, Romania; 2 National Centre for Micro and Nanomaterials & National Centre for Food Safety, Faculty of Chemical Engineering and Biotechnologies, University Politehnica of Bucharest, 313 Splaiul Independentei, 060042 Bucharest, Romania; 3 Department of Inorganic Chemistry, Physical Chemistry and Electrochemistry, Faculty of Chemical Engineering and Biotechnologies, University Politehnica of Bucharest, 1-7 Gh. Polizu Street, 011061 Bucharest, Romania; 4 Academy of Romanian Scientists, 3 Ilfov Street, 050045 Bucharest, Romania

**Keywords:** honey, bioactive compounds, biological activity, mechanisms of action, synergic effect, wound healing

## Abstract

Honey has been used for therapeutic and nutritional purposes since ancient times. It was considered one of the essential medical assets in wound healing. According to research, honeybees have significant antibacterial, antioxidant, anti-inflammatory, antitumor, and wound-healing properties. Lately, scientific researchers have focused on apitherapy, using bee products to protect and strengthen the immune system. Since honey is the most important natural product rich in minerals, proteins, and vitamins, it has been intensively used in such therapies. Honey has gained significant consideration because of the beneficial role of its antioxidant compounds, such as enzymes, proteins, amino and organic acids, polyphenols, and carotenoids, but mainly due to flavonoids and phenolic acids. It has been proven that phenolic compounds are responsible for honey’s biological activity and that its physicochemical properties, antioxidants, and antimicrobial potential are significant for human health. The review also presents some mechanisms of action and the medical applications of honey, such as wound healing dressings, skin grafts, honey-based nanofibers, and cochlear implants, as the most promising wound healing tools. This extensive review has been written to highlight honey’s applications in medicine; its composition with the most important bioactive compounds also illustrates its synergistic effect with other natural products having remarkable therapeutic properties in wound healing.

## 1. Background

The word “honey” comes from the Latin word “melem” and Greek “μελιττα”, which means “bee,” and is a product obtained through processing and manufacturing the nectar produced by bees and stored in honeycomb cells to feed the beehive population. Since ancient times, honey procurement has been the primary purpose of apiculture, and its origin appears untraceable. A Mesolithic rock was found in a cave in Valencia, Spain, dating back at least 8000 years, with illustrations of two honey hunters collecting honey and a honeycomb from a wild bee’s nest. The pictures describe the hunters carrying baskets and using a ladder or series of ropes to reach the nest [1].

In Georgia, archaeologists discovered honey remains inside an ancient tomb dating back between 4700 and 5500 years [2,3]. In Georgia, there is a custom that the buried person has a jar of honey nearby to take with them on their journey to the afterlife [4]. Documents belonging to India’s Vedic and Ayurvedic systems dating back 4000 years were found, in which the therapeutic use of honey in multiple medical applications is presented [5].

In ancient Egypt, honey had various uses, from cooking to embalming the dead. In addition, as far back as the Hellenistic period, Greek beekeepers have chosen to do pastoral beekeeping to increase their production of bee products. In other words, they move part or all of their hive to different areas depending on the species of flowering plants [6]. Furthermore, honey was used as a drug to treat various diseases dating back to 2100–2000 BC. For instance, in 384–322 BC, Aristotle described pale honey as being “good for sore eyes and wounds” [7,8,9].

Humans have used honey as food and medicine from ancient times until today due to its nutritional and health benefits [10,11]. The composition of honey varies and is connected to factors that directly affect its composition and quality, such as the bee species, floral origins, and environmental and humidity conditions [12]. The primary compound in honey is a carbohydrate, with a sugar intake of about 70–85%, especially fructose and glucose, and other components present in minor quantities, which can vary depending on the type of honey [8,13].

A significant number of studies have reported that honey has been used in traditional medicine. In addition, the role of honey as an antioxidant is essential as a hepatoprotective and cardioprotective agent [14,15,16,17,18]. Furthermore, honey has a protective effect against gastrointestinal diseases [14,19]. As mentioned above, honey is among the best natural wound healers available. The ancient Chinese, Egyptians, Greeks, Assyrians, and Romans used different types of honey to treat wounds and gut pathologies. A number of gram-negative bacteria, like *Escherichia coli*, *Salmonella* sp., *Shigella* sp., *Helicobacter pylori,* etc., display a significant or great sensitivity to the action of honey’s biologically active compounds [20,21].

Additionally, honey has anti-inflammatory [18,22,23,24] and anticancer activities on breast, cervical [25], and prostate cancers [26], as well as osteosarcoma [27]. Furthermore, honey is traditionally used as an antidiabetic [24,28], antioxidant [28], hypolipidemic agent [29] and also improves thyroid disturbances [14,30].

The review was documented using EndNote X9.3 distributed by Clarivate Analytics (Philadelphia, PA, USA) LLC as the search engine, using the following keywords: “honey” and “wound healing” with Title/Keywords/Abstract type restrictions. Based on these restrictions, 805 papers were found; thus, the findings were mainly focused on articles about synergism. Furthermore, additional searches were done to extend these data, highlight certain biological activities, explain results and mechanisms, correlate composition and activities, etc. As a consequence of this methodology, a complimentary review paper was derived, with 56 papers from the last 5 years. The first section of the review provides context for honey’s use and composition in bioactive compounds, as well as associated properties for developing wound healing. In addition, the synergic effect of honey with various products, such as cinnamon, basil, ginger, and garlic, is illustrated. Furthermore, the honey’s mechanism of action and biological activity were considered important to the research. In the end, after the mechanisms of action, attention was shifted to preclinical and clinical trials, using honey and honey-based products. Following the above structure, we consider that the main interest after showing honey’s composition and properties was to illustrate the synergic effect with natural products and the mechanism of action and biological activity of honey.

## 2. The Composition-Related Activity of Honey

The most important content of all the honey components is the bioactive compounds, including phenolic compounds. The polyphenols found in honey are correlated to botanical sources, such as pollens, nectars, resins, and different oils available to the bees. Therefore, different floral species will have distinct influences on their bioactive properties [31]. In addition, the bioactive properties of honeybees might be defined by the cerumen, a resin that stingless bees collect, combining it with saliva and beeswax to seal their hives [32].

The process is followed first by a physical transformation within the nectar in the cerumen. Afterwards, a biological change is created during the fermentation process, helped by yeasts and bacteria. Lastly, the process is followed by a chemical transformation that happens when the bees secrete enzymes from their cephalic glands during induced hydrolysis. Consequently, the physicochemical composition of the honey is influenced by the presence of phytochemicals in the cerumen [33,34,35].

**Figure 1:**
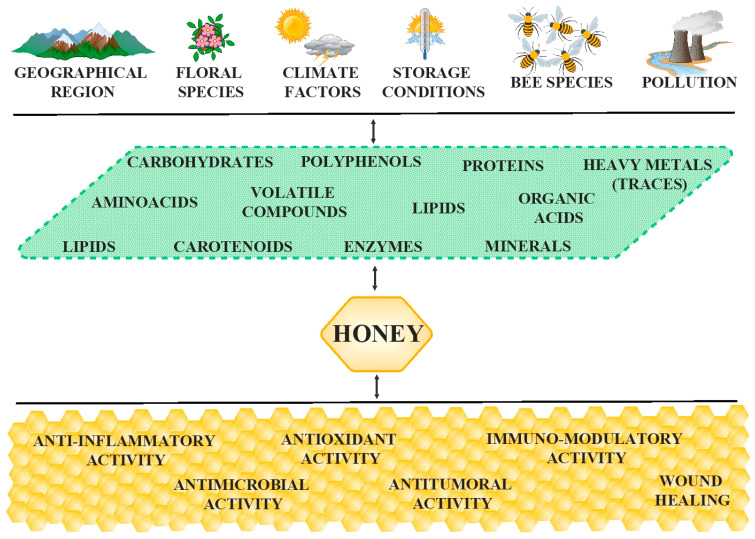
Interdependence of honey composition and biological activity by different factors. The figure was designed with ConceptDraw Diagram 16.

Further, honey contains small quantities of organic acids, amino acids, proteins, enzymes, lipids, flavonoids, and vitamins; these compounds are responsible for its biological properties (Figure 1), such as wound healing, antibacterial effects against a wide range of pathogenic bacteria [36,37], antifungal [38,39], antiviral [36,37], antioxidant [28,40,41], and antitumor [42] activities, and other skin disorders [10,36,43,44]. For example, antioxidants such as polyphenols efficiently reduce the risk of several pathologies, like cardiovascular, pulmonary (asthma), infected or chronic wounds and skin ulcers, ophthalmologic (cataracts), etc. [24,36,37,38,39,40,41,42,43,45].

The above-mentioned explanation gives reasons for the widespread use of honey obtained from commercial, artificial, and natural beehives. However, the composition of honey and its antioxidant capacity depend on flowering plants [10]. Also, its natural ingredients have shown different activities against multiple microorganisms. Its action must likely depend on the grazing grounds, the weather conditions where the bees were raised, and the natural structure of the blossom nectar [46]. It is worth mentioning that honey has an increasing effect on the levels of antioxidants, iron, and rare elements in the blood [47].

It has been reported that honey could lower cardiovascular risk in healthy patients and those with risk problems. The various parameters, such as plasma glucose, insulin, cholesterol, triacylglycerides, blood lipids, C-reactive proteins, and homocysteine, were investigated *in vivo* with natural and artificial honey; however, raw honey was used to have significant ameliorative effects on the parameters mentioned above [48]. In particular, Tualang (*Koompassia excelsa*) honey has been reported to have protective effects on memory, including enhanced morphology of memory-related brain areas, increased levels of brain-derived neurotrophic factor, reduced brain oxidative stress, increased acetylcholine concentration, and reduced acetylcholinesterase activity in brain homogenates [49,50,51,52].

Based on its chemical composition, honey can contain approximately 200 bioactive compounds (carbohydrates, proteins, enzymes, amino acids, minerals, vitamins, polyphenols, carotenoids, etc.). These substances are well known for their antioxidant, antibacterial, antithrombotic, antiallergic, anti-inflammatory, antimutagenic, anti-cytostatic, and immune-suppressive effects [44,52,53,54,55].

There is approximately 95–99% contains dry matter for all the sugar intakes. Furthermore, fructose is the most abundant, making up roughly 32–38% of its total sugar intake. In addition to fructose and glucose, there are other disaccharides and oligosaccharides, including sucrose, maltose, maltotriose, and panose. In addition, apart from sugars of all types, there are other components, such as organic acids, minerals, and trace elements, such as calcium, potassium, sodium, magnesium, phosphorus, sulphur, iron, zinc, copper, and manganese. The factors above are considered essential to various life forms having important functions in biochemical processes, as constituents of bioactive compounds, while in high concentrations, might become toxic [54,56].

There are essential vitamins, such as ascorbic acid (Vitamin C), thiamine (Vitamin B1), pantothenic acid (Vitamin B5), riboflavin (Vitamin B2), nicotinic acid (Vitamin B3), pyridoxine (Vitamin B6), biotin (Vitamin B8), folic acid (Vitamin B9), and cyanocobalamin (Vitamin B12) that are present in honey [57,58]. There are enzymes and protein constituents that play vital roles in various activities, including antimicrobial activity and calcium absorption [59]. Additionally, honey composition and biological activity depend on geographical origin, floral source, seasonal and climate factors, and production processes (see Figure 1) [54,60,61].

Antioxidant compounds, such as amino acids, enzymes, proteins, carotenoids, and polyphenols, are well credited for the beneficial properties of honey [33,62]. The flavour of honey depends on the geographical area, type of environment and storage conditions, aroma, and phenolic compounds, which depend on its botanical sources, and the bee species (*Melipona* sp. and *Scaptotrigona* sp.) involved [63,64].

A well-known fact is that phenolic compounds are responsible for honey’s antimicrobial activity, while it has been considered that its physicochemical properties and its antioxidant and antimicrobial potential are significant for human health. Several studies have revealed that the antioxidant capacity of honey also depends on the presence of flavonoids, which play a vital role in reducing oxidative stress. For instance, various flavonoids and terpenoids have been reported in multiple types of honey. In Manuka honey, pinocembrin, chrysin, pinobanksin, 8-methoxy kaempferol, luteolin, isorhamnetin, galanin, kaempferol, sakuranetin, quercetin, mangiferolic acid, and 3β-hydroxy-24-methylenecycloartan-26-oic acid have been identified [14,54,65]. Accordingly, the International Honey Commission will summarise some of the physicochemical properties of Tualang and Manuka honey, such as appearance (could be dark brown, light-dark brown, amber-brown, or colourless), moisture content (varying from 20–25%), pH (3–6), total reducing sugars (55–86%), electrical conductivity (0.49–8.77 mS/cm), and ash content (0.01–0.19 g/100 g) [14].

There is mining, as well as other industrial activities, releases many toxic metals absorbed into plants’ soil, atmosphere, and water. The honey harvested near heavy industrial sites or highways may contain high levels of harmful elements such as arsenic, cadmium, mercury, and lead, which have unknown biological functions in organisms [56,66,67]. On the other hand, a lack or reduced levels of elements in the soil, rocks, or water influence the mineral composition of different plants bees use to collect nectar. In other words, honey can serve as an excellent bioindicator of environmental pollution with heavy metals. Also, the elemental composition can indicate honey’s geographical origin and the isotopic ratio of the biologically active compounds [56,67,68,69].

Regarding composition, some minor components, such as proteins, minerals, vitamins, and phenolic compounds, play a vital role in bee activities. The study, which involves phenolic composition, includes their isolation and comprehensive characterization, which could play an essential role in elucidating their input into antimicrobial properties. Additionally, commercial standards or natural honey were used to establish antimicrobial activity [35]. The complexity of the honey matrix and phenolic compounds requires replacing the conventional, non-specific methods with other, more adequate ones. For instance, HPLC coupled with mass spectrometry offers a viable analytical alternative. The use of mass spectrometry was able to detect phenolic compounds through a high-sensitivity method and provide precise structural information [70].

Phenolic compounds are among the most chemically heterogeneous compounds produced in plants. Currently, honeybees and their products have a significant role as natural antioxidants. This is because phenolic compounds and their antioxidant properties have become the most critical features in evaluating the quality and functionality of honey [70,71].

High-performance liquid chromatography combined with a photodiode array detector or diode array detector (HPLC-PDA/DAD) is the conventional method used to determine phenolic compounds in honey [72]. Even though this technique is precise, simple, rapid, and inexpensive, additional procedures are still required. Spectroscopic technologies are the most popular for analysing the quality of honey. For example, near-infrared (NIR) spectroscopy can successfully determine the total content of honey’s phenolic, flavonoid, and antioxidant content. Additionally, Fourier transform infrared (FT-IR) and Raman spectroscopy have been used to evaluate honey quality, mainly determining sugar, moisture, and acidity. Lately, FT-IR and Raman have been effectively applied to determine the antioxidant capacity of different foods [73].

A study from Australia on extracts of stingless bee (*Tetragonula carbonaria*) cerumen determined the presence of gallic acid and pimaric acid. Moreover, cerumen is known for preventing linoleic acid oxidation, thus exhibiting antioxidant activity [74,75].

In another paper regarding *M. subnitida* honey from Brazil, gallic, vanillic, 3,3-dihydroxybenzoic, coumaric acids, and some isomers of abscisic acid (*trans-trans* and *cis-trans*) were identified [76]. Alvarez-Suarez et al. have shown the presence of ferulic acid, 2,4-dihydroxybenzoic acid, syringic acid, 3,4-hydroxybenzoic acid, ellagic acid, p-coumaric acid, *trans*-cinnamic acid, and the flavonoids, catechin, myricetin, kaempferol, quercetin, rutin, naringenin, hesperetin, and chrysin were all found in *M. subnitida* and *M. scutellaris* Latrelle honey. The HPLC-DAD-ESI-MS/ MS analysis identified 19 compounds in *M. beecheii* honey [12].

In the study [33], three bee species (*M. flavolineata*, *M. Mexicana*, and *A. mellifera*) from different Amazon regions were used. The honeybee presented 14 phenolic compounds; the significant compounds were gallic acid and quercetin. Another study [44] reported that gallic acid was the most plentiful phenolic compound in Brazilian *A. mellifera* honey. Still, p-coumaric, protocatechuic, cinnamic acid, quercetin, and myricetin were also detected in small amounts. Further, other studies show gallic acid’s effectiveness on apoptosis, prostate carcinoma, and cervical cancer cells [77].

A similar study conducted by researchers from Brazil found in honey 19 types of pollen samples, while *M. caesalpiniifolia* was found in 9 [78]. In addition, eastern Ecuadorian honey has different types of pollen (more than 14 botanical families) [79].

The study [33] reported that 16 pollen types were in honey, and the Fabaceae family was the most present. The samples with a predominantly single pollen type showed the highest total phenolic content [33].

The melissopalynological technique allows the evaluation of honey’s botanical and geographical origins. Due to floral resources, climatic fluctuations, and other native bee species, stingless bees can change their trophic position [33,76].

Accordingly, the floral source, geographic origins, storage conditions, and composition of honey’s phenolic compounds may affect the antioxidant activity of honey. Additionally, the botanical sources of nectar influence the physicochemical properties of honey. Furthermore, information regarding chemical composition confirms the variances in honey phenols’ qualitative and quantitative composition. The total polyphenols in Portugal’s rosemary, viper’s bugloss, and heather honey revealed variations from 226 µg/g for honey extract and up to 728 µg/g for natural honey. The total content of phenolic compounds in herbal honey from Romania was 20–450 µg/g [80,81].

Table 1 illustrates various countries revealing their polyphenol composition based on plant-based products. According to these data, the polyphenol content varies widely, from ~7 to over 11,000 µg/g, depending on the species and the country.

Another example of phenolic acids was presented in a study from Australia, which was relatively low and ranged from 2.13 mg/100 g in sunflower honey to 12.11 mg/100 g in tea tree honey [84]. Polish researchers have discovered phenolic acid and flavonoids in buckwheat, acacia, and honeydew honey while developing liquid chromatography and tandem mass spectroscopy analytical methods [92]. Some studies revealed that phenolic acids and phenylpropanoids showed significant antibacterial activity. The contribution of natural phenolic compounds to the nutritional quality of fruits and fruit products might play a vital role in the daily diet. Therefore, phenolic acids and phenylpropanoids could be proposed as reliable indicators of honey’s quality and authenticity [93,94].

## 3. Synergic Effect of Honey with Other Natural Products

Traditional medicine practitioner has used natural products isolated from plants and animals to treat human diseases since ancient times. As time passed, natural treatment decreased in interest in the synthetic drug industry, which provides an alternative; still, some countries rely on traditional medicine for their health care [95]. Chronic wounds and especially burns are significant to treat immediately due to the appearance of the risk of microbial infections [96]. Many healing drugs are less affordable and have partial effectiveness and toxic effects. The search for new healing agents in traditional medicine has great potential for developing effective alternative therapies [97].

Abderrahim et al. [98] reported the synergism and therapeutic properties (antimicrobial, antioxidant, and wound healing) of Euphorbia honey and *Allium sativum L*. (garlic). Regarding the antimicrobial activity, *A. sativum* exhibited better sensitivity to *S. aureus*, *E. coli*, and *C. albicans* than Euphorbia honey. Another study [90] indicated that the antimicrobial activity of *A. sativum* extracts is due to allicin, which acts by damaging DNA and RNA synthesis. Contrary to the above statement, other compounds such as diallyl disulphides, S-allyl cysteine, and diallyl trisulphide also exhibited good antimicrobial effects [99]. Moreover, due to its antioxidant and antimicrobial effects, the use of garlic to treat infectious diseases dates back centuries [100]. Furthermore, garlic contains carbohydrates, dietary fibres, enzymes, vitamins, minerals (K^+^, Ca^2+^, Na^+^, Zn^2+^, Cu^2+^, Fe^3+^, Se^2+^, Ge^4+^, Mg^2+^, Mn^2+^), polyphenols, carotenoids and at least 33 sulphur-based compounds (ajoene, allicin, alliin, allyl sulphides, allyl disulphides, allyl trisulphides, cysteine, cyclophilin, cysteine sulfoxides, cystine, diallyl sulfides, dimethyl sulphides, glutathione, disulphides, methionine, methyl sulphides, sulphates, pseudoscordinine, thiosulfinates, scordinine, trisulphides, and tetrathiol) that are responsible for poignant garlic odour and its antioxidant, therapeutic, antibacterial and anti-carcinogen effect. The bee species used in the study was *T. iridipennis* [98,101,102]. Also, Euphorbia honey presents antimicrobial, anti-inflammatory, antioxidant, and immuno-modulatory activity and stimulates wound regeneration, contributing to wound healing processes [103]. The biological properties of honey are due to its chemical composition. Honey contains mainly sugars, water, and other substances such as proteins (enzymes), organic acids, vitamins (B_1_, B_2_, B_3_, B_5_ and B_6_), minerals (K^+^, Ca^2+^, Na^+^, Mg^2+^, P^3+^, Cu^2+^, Fe^3+^, Zn^2+^ and Mn^2+^), pigments, phenolic compounds, and large particles of volatile and solid particles derived from honey harvesting [38,98,104,105,106,107,108,109,110].

**Figure 2:**
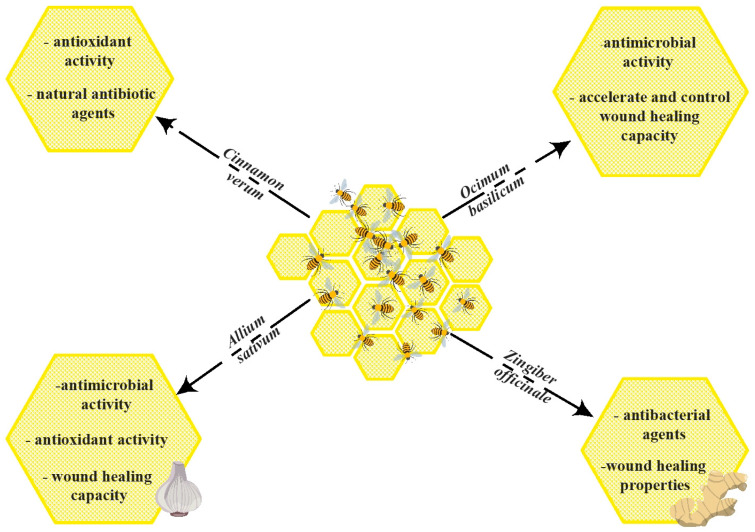
The synergic effect of honey with medicinal plants. The figure was designed with ConceptDraw Diagram 16.

Figure 2 presents the synergic effect of honey with some medicinal plants used in the studies presented in the manuscript.

In the medicinal context, synergism is the result of combining two or more components to obtain a better product with the best properties. For example, Andualem [102] presented that the mixture of Tengen honey and garlic induced a significant sensitivity of Gram-positive and Gram-negative bacteria (*E. coli*, *S. typhi*, *S. aureus*, and *Streptococcus pneumonia)*. In another study, the mixture of honey and garlic extract demonstrated better antimicrobial activity than the individual components. In contrast, it has been found that garlic extract presents better antioxidant activity than honey and their mixture [111]. Similarly, another research concluded that Euphorbia honey had a significantly higher amount of flavonoids, which led to better antioxidant activity [112].

The research has compared the burn healing capacity (epithelisation, contraction, and histological recovery) of Euphorbia honey and a mixture of honey with *A. sativum* with silver sulfadiazine and betadine solution. The data suggests that a mixture with sulfadiazine has a shorter epithelisation and contraction time compared to betadine solution and Euphorbia honey [98]. In addition, another species from the Euphorbiaceae family, *Euphorbia hirta linn*, is an important medicinal plant, and its entire plant is used to heal wounds [113,114].

In another study, the antibacterial and synergic properties of *O. basilicum* with honey were evaluated by an agar diffusion assay. Due to honey’s significant input to wound healing, acceleration and control of wound infection were demonstrated [115]. The purpose of the study was to exhibit the sensibility of several pathogenic bacteria isolated from the clinic, like gram-positive strains (*Bacillus subtilis*, *Clostridium perfringens*, *C. chauvoei*, *Enterococcus faecalis*, *S. aureus*) and gram-negative (*E. coli*, *Klebsiella pneumonia*, *P. aeroginosa*, *S. typhi*, *S. typhimirium*, *Xanthomonas campestris*) on the action of the *O. basilicum* with honey [115].

A member of the Lamiaceae family, *O. basilicum,* has been extensively grown and used in worldwide cuisine for its aromatic taste and flavour [115]. Originally, *O. basilicum* has been grown in tropical and subtropical regions, encompassing almost 150 species. The Ayurvedic system from India is considered representative of its healing properties. From a sales and business point of view, the *O. basilicum* plant’s sales have naturally increased due to the high demand, which is attributed to its ornamental nature [116]. As a plant that is used in various treatments, *O. basilicum* can help in cases of cerebral strokes, hypertension, diabetes, lipid disorders, alcohol intoxication [117], anxiety, cardiovascular diseases, headaches, nerve pains, anti-convulsing and anti-inflammatory coughs, colds, migraines, menstrual cramps, and sinusitis [118]. *O. basilicum* also has carminative, galactagogue, anti-plasmonic [119], and hemolytic activity. Additionally, it is also used in anorexia, earache, colic, kidney problems, dysentery, dizziness, gonorrhoea, insomnia, gum ulcers, piles, paralysis, and nausea [117]. A number of infections caused by various bacteria are complicated to treat because most antibiotics are ineffective due to drug resistance. In underdeveloped countries, antibiotics are costly, and the need to establish new, cheaper medications is imperious. Natural products come in handy, being recognised as safe products in numerous countries. Developing natural-based pharmaceutical products has become of great interest to researchers [117].

The studies [115] revealed that the combined effect of honey with plants, in this case, *O. basilicum,* showed synergism. It was observed that by increasing the honey concentration, the antibacterial effect was substantially enhanced. Salmah et al. [120] reported the synergism between *O. basilicum* and honey against cutaneous wound healing in rats. The results of the study showed that adding plant extracts improved the healing process. In a similar study, it was discovered that honey has superior antimicrobial activity against *Clerodendrum myricoides* when compared to *C. albicans* [115].

This synergic antibacterial effect can be highly efficient in treating infested injuries and can be utilized as a prospective antimicrobial. Furthermore, by knowing the mechanisms and identifying the components within the honey or basil oils, additional studies must be done to find the response for the synergic effects. The method of assigning the exact mechanism of synergism between honey and *O. basilicum* can be carried out to treat infected wounds and other bacterial infections [115].

Rezvani et al. [121] also investigated the synergic effect by evaluating the antibacterial activity of honey and cinnamon against *S. mutans*. In addition, studies have shown that *S. mutans* is the primary bacteria causing dental caries. The study aimed to develop antibacterial agents that affect *S. mutans* and could inhibit plaque development on tooth surfaces. The interest of researchers in alternative medicine, strictly in using herbal extracts, has increased recently. Further, numerous studies have reported substantial antibacterial activity for plants and natural antibiotics against *S. mutans* [122,123,124]. In addition, the antimicrobial capacity of honey has been intensively studied. Still, no data are available regarding the effects of the mixture of cinnamon and honey against cariogenic bacteria [121].

The combination of different plant extracts against the target bacteria would ensure the exposure of the pathogens to chemicals, which could lead to totally intensified activity [125,126]. Therefore, it was discussed that the combined treatment with honey and some plants displayed a significant synergism against bacteria compared to their natural extracts [127]. For example, honey and cinnamon extracts are powerfully effective against *S. mutans*, and it can be implied that their combination would be more productive [121].

Furthermore, mixing another plant like ginger has proven honey’s synergistic antibacterial properties. Ahmed et al. [128] discussed this aspect, including the synergism of honey and ginger against bacterial strains like *E. coli* and *S aureus*. Similarly, the study also demonstrated that the extracts of honey and ginger have the potential to serve as antibacterial agents for drug-resistant bacterial strains. In conclusion, it can be stated that neither honey nor ginger hurts human tissues. They could be safely integrated into oral products to prevent caries formation. Additionally, the synergism of both agents would have a considerable therapeutic effect against *S. mutans* [129]. Table 2 presents some information regarding the synergic effect of honey on other compounds.

## 4. Mechanisms of Action and Biological Activity of Honey

**Figure 3:**
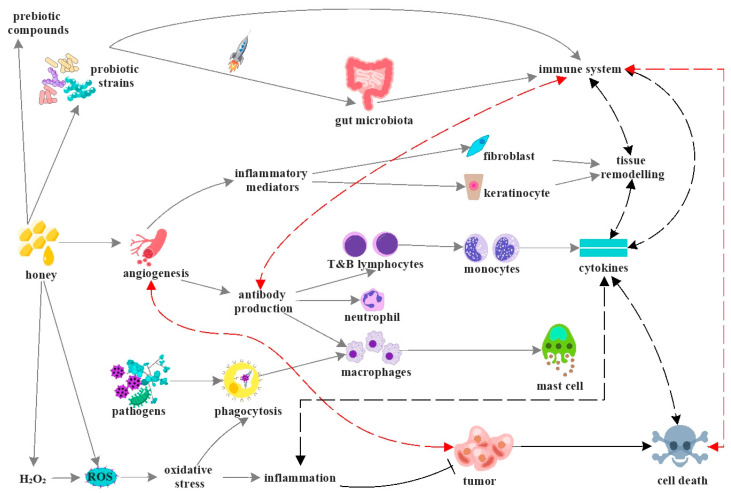
Graphic representation of the immunomodulatory mechanism of honey. The figure was designed with ConceptDraw Diagram 16.

The present section refers to the biological activity of honey and its mechanisms of action due to its bioactive constituents [130]. This product can be an immunomodulatory agent due to its anti-inflammatory and antioxidant activity [131,132], and it stimulates the proliferation of T and B lymphocytes and phagocytosis. Also, honey regulates the production of proinflammatory cytokines (tumour necrosis factor α ~ TNF-α, interleukins 1β and 6 ~ IL-1β, IL-6) [133,134]. The immunomodulatory activity of honey is due not just to polyphenols but also to the sugar held [135,136]. Moreover, this apitherapeutic product contains prebiotic compounds (such as non-digestible carbohydrates and polyphenols) that improve the gut microbiota. Furthermore, honey contains probiotics, which play an essential role in gut health. As seen in Figure 3, honey develops an interdependence and synergic effect between all biological activities and boosts the immune system and health [130,137,138,139].

Kowalska et al. [140] demonstrated for the first time that using bee products encapsulated with arabinoxylans isolated from the rye bran could significantly ameliorate the inflammatory response in lipopolysaccharide (LPS)-treated RAW 264.7 macrophages, decreasing the secretion of IL-6, TNF-α and nitric oxide (NO). For the encapsulated core, it has been made with honey and royal jelly. Moreover, the honeydew honey microcapsules exhibited significantly more antioxidant activity than native honeydew honey. In the case of the royal jelly, it was quite the opposite. The antioxidant activity was not different from the native royal jelly. Therefore, the authors showed that using bioactive heteropolysaccharide carriers to encapsulate honey and royal jelly to develop innovative platforms with controlled release of bioactive compounds with potential immunomodulatory properties.

## 5. Antimicrobial Activity

Kwakman et al. [141] suggested that the antibacterial activity of honey against *B. subtilis*, *S. aureus* MRSA, *E. coli*, ciprofloxacin-resistant *P. aeruginosa*, and vancomycin-resistant *E. faecium* depends on H_2_O_2_, sugar, methylglyoxal, and bee defensin-1. The mechanisms of action depending on the type of honey and strains. For example, in another study, Kwakman et al. [142] showed that the antibacterial activity of manuka honey against *S. aureus* and *B. subtilis* depends on methylglyoxal and several unknown factors or compounds (such as polyphenols). In addition, the antimicrobial activity is due to the low pH, osmotic stress (high sugar content), and the presence of enzymes that produce hydrogen peroxide [143].

Henriques et al. [144] reported the bactericidal activity of manuka honey against *S. aureus* strains. According to TEM analysis, structural changes were observed in honey-treated cells. Honey determined an inhibition in cell growth, altered the cell cycle, and accumulated with fully developed septa at cell division without separating. In another study [145], enlarged cells containing septa in methicillin-resistant *S. aureus* were observed when treated with manuka honey, which suggested the interruption of cell division. Moreover, this study shows that these changes were due to other antibacterial compounds, not sugars or methylglyoxal.

In particular, manuka honey inhibits *P. aeruginosa* by destabilizing the cell membrane and downregulating a structural protein (OprF), which maintains the cell shape and stability [146,147]. The SEM and TEM images indicate the presence of artefacts and abnormal cells in *P. aeruginosa,* which suggests cell disruption and lysis. The honey has a moderate antibacterial activity on *P. aeruginosa*, doesn’t act with a similar mechanism as for *S. aureus*, and produces different structural changes [146]. A recent study [148] shows that manuka honey’s antibacterial activity against *P. aeruginosa* has multiple pathways. Thus, Bouzo et al. [148] demonstrate that the antimicrobial activity of Manuka honey cannot be explained only by the methylglyoxal presence. Manuka honey induces several transcriptional changes and affects many biological processes (plasmid, antibiotic resistance, adaptation, and protection are upregulated). Furthermore, other functions are overexpressed by honey, such as the transport of molecules, RNA processing and degradation, nucleotide biosynthesis, membrane proteins, metabolism, DNA replication, cell wall degradation, biosynthesis of cofactors, etc. Protein secretion, fatty acid metabolism, amino acid biosynthesis, and metabolism are downregulated by manuka honey’s action.

The manuka honey damages the cytoplasmatic membrane by depolarisation. As such, the collapse of the proton motive force and membrane permeabilization can be another key to the antibacterial activity of manuka honey [148]. The physiological changes associated with membrane polarization and integrity are presented in a recent study [149]. Similarly, avocado, chestnut, and polyfloral honey induce the same effects on *S. aureus* and *E. coli*, especially membrane damage.

**Figure 4:**
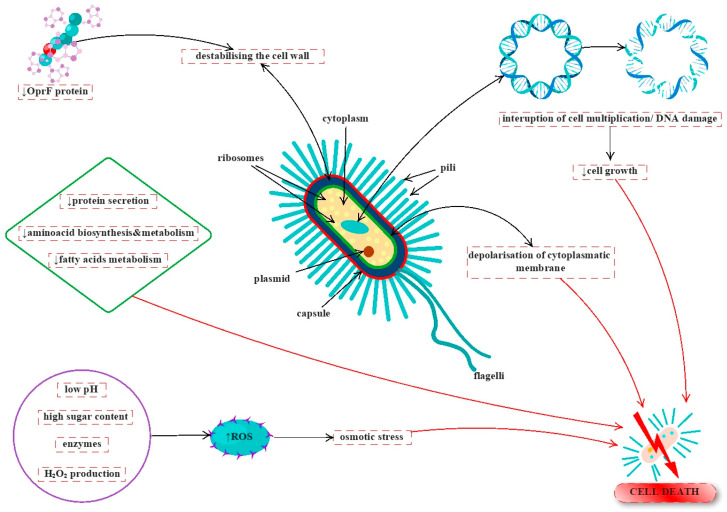
Antimicrobial mechanisms of honey. The figure was designed with ConceptDraw Diagram 16.

The relationship between mechanisms of action and activity is correlated with honey type, chemical composition, concentration, physical properties (pH, water content, etc.), and storage conditions [149,150], as highlighted in Figure 4.

Maeda et al. [129] have conducted a study regarding the antibacterial activity of honey against community-associated methicillin-resistant *S. aureus* (CA-MRSA), describing the therapeutic effect of honey on skin and soft tissue infections. Lately, it has been observed that methicillin-resistant *S. aureus* (MRSA) has appeared within healthcare facilities, particularly hospitals. Additionally, it has been reported that *S. aureus* MRSA occurred among healthy individuals without hospitalisation. They are very different from healthcare-associated MRSA (HA-MRSA). This community-associated methicillin-resistant *S. aureus* (CA-MRSA) is usually associated with severe skin and soft tissue infections, particularly in young, healthy individuals and those with no risk factors for the acquisition of *S. aureus* HA-MRSA. Further research still needs to be conducted to indicate that this antimicrobial activity has clinical application [151].

In other research, Ghramh et al. [152] also considered the antibacterial potential of three honey samples collected from the Saudi region against pathologic bacteria. The purpose of the study was to evaluate the antibacterial activity of Sider (*Ziziphus spina-christi*), Dharm (*Lavandula dentata*), and Majra (*Hypoestes forskaolii*) honey samples collected from the Asir region of Saudi Arabia and investigate *in vitro* the antibacterial activity of these honey samples against *E. coli*, *Proteus mirabilis*, *S. aureus*, *S. flexneri*, and *S. epidermidis.* Results confirmed that Dharm and Sider honey samples showed better antibacterial activity than majra honey. Further, Saudi honey can be considered a promising future antimicrobial agent. It should be investigated as an alternative for managing resistant bacterial pathogens [153,154].

The increasing resistance to antifungal drugs has prompted the need to evaluate new antifungal compounds with fewer side effects. Drug resistance and honey have attracted attention mainly for their potential antifungal effects. This study presents the antifungal activity of four types of honey from Algeria, which were tested against pathogenic yeasts such as *C. albicans* and *Rhodotorula* sp. Among commensal organisms, *C. albicans*, a polymorphic fungus, was investigated due to the significant damage caused by inhabiting the oral, vaginal, and gastrointestinal mucosa. The results of *in vitro* studies confirmed that it could inhibit the growth of many species of *Rhodotorula* sp., but no effect was reported on *C. albicans* [39]. Similar studies have reported the behaviour of C. albicans at a microscale level after treatment with Euphorbia *hirta* L. leaf extract. Results found multiple irregularities concerning morphology, lysis, and yeast cell disintegration. The control cultures showed normal morphology for *Candida* sp., with uniform density, a structured nucleus, and an endomembrane cytoplasm with a regular, intact cell wall. In conclusion, the study suggested innovative approaches to developing appropriate anticandidal agents for human infections [155,156]. Additionally, *C. albicans* and *Rhodotorula* sp. were investigated due to venous catheter-associated illness and fungemia. The results show that using a specific antifungal therapy may increase the survival chances of hospitalized patients and, most importantly, reduce the cost of health care, morbidity, and mortality [157,158]. Regarding the *in vitro* antifungal activity of lavender honey, Estevinho et al. [38] observed that honey stops the growth of *C. albicans*, *C. krusei*, and *Cryptococcus neoformans*. The authors reported that the biological activity of lavender honey was typically credited to the phenolic compounds. The phenolic antimicrobial mechanism is related to their potential to denature proteins [38].

In recent times, one of the most crucial challenges in medicine is fighting antibiotic resistance against a broad spectrum of infections caused by different pathogenic agents. Due to intensive progress and scientific advances, research has given birth to nanotechnology, an intelligent and innovative alternative that could solve problems related to microbial resistance. Consequently, nanomaterials and nanoparticles are successfully used in various applications in medicine and pharmaceuticals [159]. Silver nanoparticles synthesised using plant extracts have been chosen because they have promising applications in fields like drug and gene delivery, biological sensors, catalysts, electronics, energy storage, antimicrobial protection, and biomedical treatments [160]. Therefore, it is used as a cytotoxic agent against cancer cells [161]. In recent years, honey has attracted attention as a mediator in silver nanoparticles’ green synthesis to treat various health disorders. Honey is a natural product with an active function in the inhibition process for many pathogenic organisms because it is described as having essential active compounds such as flavonoids, glycosides, and phenolic acids. As in many studies that confirmed the efficacy of silver nanoparticles against many perturbing factors, silver nanoparticles were produced by using Tualang honey in Malaysia as a stabilizing agent [162]. This study reveals the potential of honey from two different floral sources (*Ziziphus spina-christi* and *Acacia gerrardii*) as biogenic mediators for silver nanoparticle synthesis to evaluate their antioxidant, cytotoxic, and antibacterial properties. This ultimately indicates that synthesizing silver nanoparticles using bee’s honey is an effective agent in some biomedical applications. Moreover, Ag has proven to suppress microbially and cell growth; biogenic AgNPs were cytotoxic against HepG2 cells and antibacterial against *S. aureus*, *P. aeruginosa,* and *E. coli*. AgNPs showed a high antioxidant capacity with repercussions on suppressing microbial and HepG2 cell growth [163].

The high sugar content of honey influences the growth of most microorganisms, but even diluted honey solutions still have important antimicrobial properties that are often higher than those of sugar solutions. Since the 1960s, H_2_O_2_ production has been an essential aspect of the biological activity in honey [164]. H_2_O_2_ is produced when glucose oxidase, secreted from the pharyngeal glands of the bee and presented in honey, reacts with water. While glucose oxidase may vary in different kinds of honey, many types of honey are generated. H_2_O_2_ is at the proper level to kill microorganisms. A specific *Leptospermum* honey from Australia and New Zealand exhibits “extra” antimicrobial properties due to the volatile parts of the floral compounds [165]. This mystery regarding the “extra” antimicrobial activity of *Leptospermum* honey has not been identified yet [166,167]. The research on the honey samples revealed unique, unusual antibacterial activity due to newly identified compounds. The study on broad-spectrum bacteria showed that antibacterial activity has the same effectiveness against antibiotic-resistant pathogens. Results also confirmed that honey positively affects tissue growth and stimulates wound healing [168].

## 6. Antioxidant Activity

An antioxidant is a substance that, at low concentrations, interrupts or prevents substrate oxidation and acts through mechanisms such as single electron transfer, hydrogen atom transfer, or chelating transition metals [169].

In the organism, oxidative stress is a complex process represented by the imbalance between the production of free radicals and the capacity to eliminate them with endogenous (enzymes, bilirubin, albumin) and exogenous antioxidants (phenolic compounds, carotenoids, vitamins, etc.). The reactive oxygen species (ROS), such as superoxide radical anion, hydrogen peroxide, etc., are the promoters in the metabolic processes. Any excess ROS can determine several pathologies [170]. Honey can be considered an exogenous antioxidant due to its chemical composition, especially for phenolic compounds [171].

The honey’s mechanisms of antioxidant activity could involve glutathione reductase (GR), vitamin C (Vit. C), beta-carotene, uric acid, hydrogen donation, metallic ion chelation (MIC), or free radical removal (FRR). Additionally, honey reduces ROS and stimulates biomolecules (nucleic acids, proteins, carbohydrates, lipids, etc.) [28,41]. Furthermore, the antioxidant activity of honey depends on the botanical source, and darker honey has more antioxidants than lighter honey [172,173,174]. In Table 3, the effects of honey administration can be seen.

Quantifying honey’s quality, composition, and antioxidant capacity must be considered, which depends entirely on the floral source from which the nectar was collected, season, processing, and environmental factors [182]. These factors may also influence the composition of the honey and its antioxidant capability. It is well known that free radicals cause oxidative stress, which might lead to many disorders such as cancer formation, inflammation, ageing, the progression of diabetes, cardiovascular diseases, weakening of the immune system, degenerative diseases of the nervous system, heart and lung diseases and cataracts also [60]. Living in the age of technology has encouraged humans to consume healthier foods, especially those rich in natural antioxidants, which may prevent and treat chronic disorders. These natural antioxidants can fight against free radicals, and honey is the most appropriate natural food rich in antioxidants [76,183].

Honey has antimicrobial, antiviral, antiparasitic, anti-inflammatory, antimutagenic, anticancer, and immuno-suppressive actions [184]. The therapeutic techniques using bee products to protect and strengthen the immune system are called apitherapy. Since honey is the most important natural product rich in minerals, proteins, vitamins, phenolic compounds, and other essential acids, it has been intensively used in these techniques [185]. The main goal of this study was achieved, and the antioxidant capacity of several mono-floral honeys from Turkey has been proven [60].

## 7. Anti-Inflammatory Activity

Inflammation is the immune system’s response triggered by dangerous factors, such as pathogens, tissue injury, toxic compounds, irradiation, etc., which can induce acute or chronic inflammatory processes [186]. In response to tissue damage, the organism initiates a chemical signalling cascade, which activates leukocyte chemotaxis and cytokine production [187]. Otherwise, inflammation is a defence mechanism for the organism. It is a critical element in the pathological progression of many diseases [186,188]. Also, the human body’s oxidative stress and inflammation processes are associated with multiple signalling pathways [189]. Moreover, ROS production leads to inflammation and the release of cytokines in the damaged tissue [190].

The anti-inflammatory activity of honey is due to the phenolic compounds [191]. The bioactive compounds of the honey act by downregulating the inflammatory transcription factors or by controlling the production of cytokines and inflammatory mediators: prostaglandin E_2_, cyclooxygenase-2 (COX-2), tyrosine kinase, and ornithine decarboxylase (ODC) [131,132,136]. Similarly, honey stimulates the antioxidant capacity of cells. It inhibits the inflammatory response by downregulating nuclear factor-kB (NF-kB), NLR3 inflammasome, and mitogen-activated protein kinases (MAPK) signalling and upregulating AMPK, nuclear factor erythroid 2 (Nrf2), antioxidant response element (ARE), heme oxygenase-1 (HO-1), and interleukin-10 (IL-10) [192].

A recent study identified a new bioactive compound in honey: vesicle-like nanoparticles (H-VLNs), which contain proteins, lipids, and small-sized RNAs [193]. The administration of H-VLNs inhibited the activation of NLRP 3 inflammasome and ameliorated the mice’s inflammation and liver damage. Additionally, the H-VLNs suppressed the formation and activation of NLRP 3 inflammasome, all downstream processes like generation of Casp1, and secretion of cytokine IL-1β and IL-18 [193].

## 8. Antitumor Activity

The biological properties of honey also include antitumor activity. Honey can inhibit the growth and proliferation of tumoral cells by regulating the cell cycle, activating mitochondrial pathways, inducing apoptosis, permeabilizing the outer membrane, regulating ROS production, reducing inflammation, and modulating angiogenesis and insulin signalling [194,195]. Honey can be an antitumor agent due to the polyphenols and carbohydrates it contains [136,194,195].

Cell death involves three phases: the induction phase, the effector phase, and the degradation phase [196]. Additionally, cell death can be classified into three types: type I, or apoptosis, which shows cytoplasmatic shrinkage, nuclear disintegration, chromatin condensation, plasma membrane bleeding, and the formation of apoptotic bodies that are degraded by lysosomes; type II, or autophagy (cytoplasmatic vacuolisation and lysosomal degradation), and type III, or necrosis, which is characterized by the absence of phagocytosis and lysosomes [197,198]. In general, apoptosis can be initiated by an extrinsic pathway (death receptor) that acts within caspase 8 or by an intrinsic (mitochondrial) way through caspase 9. Specifically, caspases 8 and 9 are converged in caspase 3, which performs death substrates. Moreover, cell death is characterized by overexpression of B-cell lymphoma protein 2 (BCL_2_) and by an inhibitor of apoptosis (IAP) protein [199].

**Figure 5:**
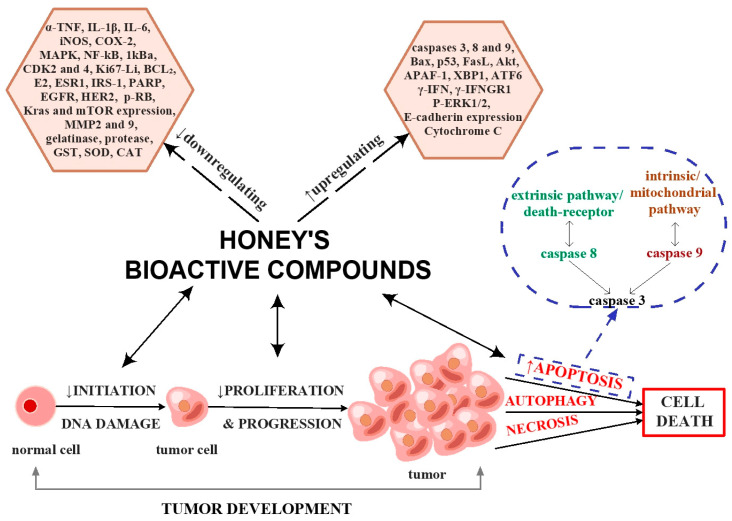
Antitumor effects of honey’s bioactive compounds. The figure was designed with ConceptDraw Diagram 16.

Honey induces apoptosis by upregulating or stimulating caspases 3, 8, and 9, Bax, p53, apoptotic protease activating factor 1 (APAF-1), γ-interferon (γ-IFN), γ-interferon receptor 1 (γ-IFNGR1), altered P13 kinase (Akt), fatty acid synthetase ligand (FasL), extracellular signal-regulated protein kinase (P-ERK1/2) expression, E-cadherin expression, antioxidant response element 6 (ATF6), X-box binding proteins 1 (XBP1) and cytochrome C. Additionally, honey acts by downregulating/ inhibiting nuclear protein Ki67 labelling index (Ki67-LI), BCL_2_, iNOS, COX-2, estrogen (E2), estrogen receptor 1 (ESR1), insulin receptor substrate (IRS-1), poly(ADP-ribose) polymerase (PARP), α-TNF, IL-1β, IL-6, gelatinase, protease, MAPK, NF-kB, an inhibitor of kappa B (1 kBa), cyclin-dependent kinase (CDK2 and 4), human epidermal factor receptors (EGFR, HER2), p-retinoblastoma (p-RB), Kras gene expression, mammalian target of rapamycin (mTOR) gene expression, malondialdehyde (MMP 2 and 9), glutathione S-transferase (GST), superoxide dismutase (SOD) and catalase (CAT) [28,194,200,201,202,203,204,205,206]. A summary of the mode of action of honey’s bioactive compounds as antitumor agents is represented in Figure 5.

## 9. Wound Healing Activity

**Figure 6:**
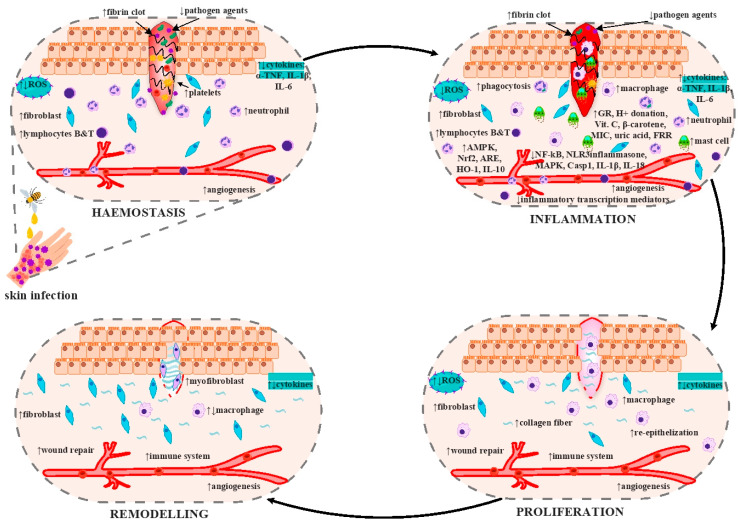
Potential of honey as a wound healing agent. The figure was designed with ConceptDraw Diagram 16. “↑”-high and “↓”-low quantity.

The wound-healing process is complex, and the mechanisms involved have multiple pathways. As explained above and represented in Figure 6, honey inhibits the growth of pathogens, stimulates the immune cells, regulates cytokines and ROS production, and stimulates the wound repair processes by accelerating re-epithelization. Furthermore, honey’s bioactive compounds accelerate collagen matrix production [207].

Figure 6 describes the effects of honey on the stages of wound repair and their important cellular mechanisms. Wound healing is usually divided into 4 steps: haemostasis, inflammation, proliferation, and remodelling. During the first step, in the haemostasis phase, activated platelets are blockers to impede bleeding and help fibrin matrix formation. The two most crucial roles of platelets are to inhibit bacterial infection and to recruit immune cells [208,209]. The inflammation happens as a defence mechanism against pathogenic wound invasion. After the injury, neutrophils are engaged in removing necrotic tissue from the wound, releasing ROS and other enzymes. Like neutrophils, macrophages also eliminate necrotic cellular remains and promote inflammation by releasing ROS, cytokines, and growth factors [210,211]. During the proliferation phase of wound healing, keratinocytes migrate onto the wound to restore the epidermal layer. Fibroblasts replace the previous fibrin matrix with granulation tissue. Still, most significant new blood vessels are developed during angiogenesis and proliferative wound healing. Macrophages and regulatory T cells are essential and vital for wound healing [212,213,214,215]. The remodelling process is characterized by anti-inflammatory macrophages, myofibroblasts, fibroblasts, collagen fibrils, and keratinocytes, which restore wound healing [215,216].

The bodies of children are more vulnerable to injuries and wounds, so they are susceptible to nasty infections. Therefore, there should be consideration of proper antiseptic modalities to prevent and treat diseases and disorders. New antibiotics must be developed, but resistance can be generated quite quickly, so many researchers have become reticent about their development [168,217,218]. As we all know, honey is used for its antimicrobial and wound-healing properties. Still, medical-grade Manuka honey (MGH) was developed to provide insurance and efficacy for clinical applications. Additionally, MGH must fulfil some requirements, such as killing endospores, being pollutant-free (pesticides, heavy metals, etc.), and having rigorous quality processing and storage standards and regulations. Further, MGH acts as an antimicrobial agent and decreases the risk of developing antimicrobial resistance. It must be noted that all of the honey’s constituents play essential roles in antimicrobial activity. Still, significant antimicrobial activity is shown where the higher concentration of polyphenols is presented. Flavonoids, acting as antimicrobials, induce diverse mechanisms such as DNA inhibition, cytoplasmic membrane, and energy metabolism [219]. The most important feature of the MGH is preventing pathogens from invading the infection. Finally, MGH is also confirmed to be successful in its application to wounds such as traumatic injuries, burns, hematomas, pressure ulcers, diabetic foot ulcers, lacerations, and skin tears [220,221,222,223].

Next, another example of honey’s biological and therapeutic properties in wound healing will be presented. After many years of studying honey for its medical applications, its essential role in wound healing has been clarified. Additionally, after reviewing honey’s mechanisms of action and therapeutic properties in wound healing, clinical trials are the next step in recovery. Laboratory studies have compared honey with hydrofiber silver or silver sulfadiazine; still, honey was more effective in eliminating microbial contaminants and promoting re-epithelialization [37]. For example, a study explored the systemic pathology of diabetic wounds with the beneficial help of using honey during the regeneration process [224]. Further, animals are also crucial for wound healing; as a result, the first study was reported in Algeria regarding the use of honey for cutaneous wound healing in horses [225].

The presence of the organic compound 5-hydroxymethylfurfural (HMF) may contribute to honey’s toxicity or not; therefore, its presence in honey or other foods must be thoroughly investigated [226]. However, HMF is not naturally formed in honey or other food products [227]. While honey contains water, sugars, and other compounds, due to inadequate storage conditions (high temperature, metallic containers, etc.), HMF could be formed. The honey sugar degradation at high temperatures through the Maillard reaction led to HMF production [228]. Therefore, to ensure honey’s freshness and safety parameters, there have been standards and regulations that set the maximum limit for HMF in honey at 40 mg/kg [229,230]. Nevertheless, HMF presence in honey due to heat treatment or extended storage may enhance HMF production, which might have negative effects on human health, such as mutagenicity, hepato- and renal toxicity, chromosomal aberrations, and irritation of the mucous membranes of the skin, eye, and respiratory tract [231,232].

Although there is new research on HMF’s widely positive effects on humans, such as anti-inflammatory [233], antioxidant [234], and antiallergic [235] are some effects that highlight the negative and positive nature of HMF on human health. Another important aspect that has to be investigated is the HMF effect on the honeybee. Among the studies presented in the literature, authors established that using HMF-food supplements has caused dysentery, intestinal ulceration, and even bee death [236]. Therefore, in addition to the presented information on the negative effects of HMF on humans and honey and the further use of honey in wound healing applications, extensive research must be considered to establish an adequate formation and usage of HMF in honey [226].

## 10. Preclinical and Clinical Trials Using Honey and Honey-Based Products

As already stated in this review, honeybees are effective agents in wound healing. Thus, it is used in the development of wound-healing dressings. It has started to be intensively harvested in developing countries, mainly used in wound dressings. The Faculty of Medicine from Egypt has conducted a clinical test [237] that implied the utilisation of honey dressings on patients with infected diabetic foot wounds. The treatment involves the application of honey to the patient’s wounds for three months until healing occurs when the grafting treatment fails. There were significant changes noted within the foot wounds. The bacterial load was reduced after the first week of treatment, and complete healing was observed in 43.3% of ulcers. Still, it has to be mentioned that failure was observed in 6.7% of cases of ulcers. After finishing the trials, studies demonstrated that honey dressings are a good candidate for the treatment of patients with diabetic foot wounds. Most importantly, it has proven safe, cost-effective, and clinically effective.

Another experiment [238] also proved honey’s medicinal purpose. The study uses honey for split-thickness skin graft fixation, primarily due to its adhesive effects. There were eleven patients with diverse skin graft diagnoses included in the study. Based on the results, it has been shown that honey is a very effective agent used for split-thickness skin graft fixation, and no infection or graft rejection was observed. Since honey is a natural agent, it can be successfully used in all skin grafts to fix split-thickness skin grafts.

Lately, honey has dramatically impacted all medical fields, especially in apitherapy, which treats disorders with the help of bee products, including honey. The healing properties of honey have been known since antiquity. Additionally, its various phytochemical compounds, rich in phenols, contributed to its biological properties. A number of studies presented the idea that antibacterial properties are due to the levels of hydrogen peroxide, glucose oxidase, and catalase [21]. Despite that, some studies have shown that the antibacterial activity of Manuka honey is attributed to methylglyoxal and not hydrogen peroxide [239].

Moreover, in addition to the antibacterial and antioxidant action, honey displays extensive therapeutic properties, for example, antiulcer, anti-atherogenic, antiviral, and anti-inflammatory [240]. Based on the evidence that somatic mutation promotes cancer formation and is responsible for superoxide anion radicals and inflammation, the anticancer effect of honey has recently been investigated. The studies made in this direction on animal models and clinical trials have demonstrated that honey has anticancer activities. Cancer is deadly, and treatments depend on the cancer stage and, unfortunately, are not 100% successful or effective. It has been studied that the right approach against cancer should be via chemoprevention. This new concept involves a balanced diet and a healthy life with daily controlled consumption of honey, reducing cancer risk. Additionally, honey as a chemo-preventive agent is defined as using natural or synthetic compounds to eliminate the risk of developing or reoccurring cancer [241]. H_2_O_2_ has a double feature in cancer development. First, the higher amounts of H_2_O_2_ are responsible for the alterations of tumour cells. On the other hand, H_2_O_2_ can induce apoptosis of tumour cells, which modulates the activity of the anticancer drug [242]. Furthermore, *in vitro* and *in vivo* studies have shown that honey’s antiproliferation activity is due to cell cycle arrest, which includes a synergy with antitumour drugs [243].

Another promising application was the fabrication of pomegranate or honey nanofibers for antibacterial wound dressings. The tests consisted of honey, pomegranate peel extract, and bee venom samples used with polyvinyl alcohol to develop a novel nanofibrous wound dressing [244]. The nanofibrous scaffolds present better properties than conventional dressings due to their larger surface-to-volume ratio, higher porosity, and tiny pore size. These advantages include better wound permeation and prevention from further infection [244]. Additionally, the use of honey in wound healing was due to its antimicrobial and anti-inflammatory properties [245]. It has been stated that the combination of honey and PVA, a biocompatible polymer, seems promising, and honey/PVA-based gels have better biocompatibility for creating burn-care dressings [246]. It has been demonstrated that pomegranate and its peel have great potential due to their anti-inflammatory and anti-infective effects. Pomegranate juice and its peel have significant amounts of polyphenolic compounds such as ellagic and gallic acids, flavanols, anthocyanins, catechins, and procyanidins. The peel possesses more polyphenolic compounds than pomegranate juice, thereby having promising wound-healing potential. Therefore, after displaying the essential properties of pomegranate and peel extract, it can be concluded that it could be used in designing wound dressings [247].

Furthermore, all honey-based products derived from beekeeping, pollen and propolis are being used for their nutritional and medicinal purposes. Still, propolis has special interest due to its antimicrobial and antioxidant properties, which are beneficial in developing honey-based healing dressings [126,191]. Another interesting natural product included in many wound dressings was bee venom. Venom has been the focus of many research groups for its potent therapeutic effect in various pathological conditions ranging from pain, rheumatic arthritis, skin diseases, and tumours. Similarly, bee venom is a promising candidate for wound healing due to its powerful antibacterial effect and potent anti-inflammatory properties [248,249]. In a study [250], pomegranate peel powder extract and bee venom were loaded within honey/PVA nanofiber scaffolds to test their wound healing activity in an animal model using two types of honey: manuka honey and lyophilised multiflora honey powder. The pomegranate peel powder extract shows significant antioxidant and antibacterial effects. The results demonstrated that it also promoted wound healing, collagen regeneration, fibroblast infiltration, vascularisation, and epithelialisation. In addition, it was reported that honey-based nanofibers presented strong antibacterial activity against gram-positive and Gram-negative organisms. It was noted that manuka honey was more effective than lyophilised honey against *E. coli*. However, excellent antibacterial activity was also observed in lyophilised honey, suggesting a possible synergic effect between honey and pomegranate peel powder that led to strong antibacterial activity against *E. coli*. Cytotoxicity tests have shown that all scaffolds had ~100% cell viability, indicating that the nanofibrous dressings have no significant cytotoxicity against skin cells. In conclusion, results indicated that Manuka honey/ pomegranate/ bee venom nanofibers are promising instruments for wound healing [250].

The use of honey in wound care has become more and more critical due to its proven beneficial properties in wound healing and its antibacterial and antibiofilm properties. Costeloe et al. [251] presented the experience of medical-grade honey with successful outcomes in treating a cochlear implant. The results demonstrated that the patients had complete wound closure without surgical reconstruction.

## 11. Conclusions

Over time, biomedical sciences have shifted attention toward honey due to its therapeutic effects and biological properties. Recent findings confirmed that honey has antimicrobial, antioxidant, and anti-inflammatory activities and fights against diseases from wounds to cancer. Additionally, it has been proven that honey’s beneficial use in medical applications is responsible for the presence of flavonoids and vitamins within it. The review highlights how the most important content of honey is attributed to the bioactive compounds, which include constituents such as phenolic compounds, amino acids, proteins, enzymes, lipids, flavonoids, and vitamins and are responsible for its biological properties such as wound healing, antibacterial, antifungal, antioxidant, and antiviral activities. The antioxidant efficacy of honey is credited to its content of amino and organic acids, proteins, enzymes, carotenoids, polyphenols, and especially its flavonoids and phenolic acids. Special attention was paid to the synergic effects of the interaction of honey with other natural products containing bioactive compounds that possess therapeutic properties and can assure a synergic result. Furthermore, the other natural products described in this review that can be synergic with honey are garlic, cinnamon, and basil, which have antibacterial, antioxidant, and antimicrobial activities. For example, basil and garlic have good wound-healing properties, while cinnamon fights against bacterial infections. On the other hand, garlic’s antioxidant and antibacterial properties have proven their usefulness in treating infectious diseases. Another natural compound described in this review was basil, which showed significant synergism with honey against cutaneous wound healing in animals. The review also presented the synergic effect of honey with cinnamon against *S. mutans* bacteria. Several studies in this direction have reported a strong antibacterial effect against *S. mutans* that could prevent plaque formation on tooth surfaces. As a result, research on natural products considers synergism between the plant ingredients to benefit the maximum therapeutic efficiency. Additionally, the review focused on the most critical applications of honey in the medical field, especially in wound care. In conclusion, it can be stated that honey could be considered a promising instrument with significant beneficial applications in wound healing dressings. Therefore, understanding the therapeutic effects of honey and its essential bioactive compounds and molecular mechanisms may provide significant insights for conducting novel approaches with applications in future clinical trials. 

## Figures and Tables

**Table 1:** Polyphenol composition in various countries.

No.	Polyphenols [µg/g]	Plant-Based Food	Species Name	Country	Reference
1	226.00	Rosemary plant	*Rosmarinus officinalis*	Portugal	[80]
2	406.00	Viper’s Bugloss Plant	*Echium vulgare*	Portugal	[80]
3	728.00	Heather plant	*Calluna vulgaris*	Portugal	[80]
4	20.00	Acacia plant	*Robinia pseudoacacia*	Romania	[81]
5	128.00	Lime plant	*Tipia* sp.	Romania	[81]
6	450.00	Honeydew	Mountain multi-flora	Romania	[81]
7	131.00	Apiaceae fruit	*Lagoecia cuminoides*	Greece	[82]
8	716.00	Asteraceae herb	*Artemisia arborescens*	Greece	[82]
9	54.00	Asteraceae leaves	*Taraxacum officinale*	Greece	[82]
10	802.00	Caprifoliaceae flower	*Sambucus nigra*	Greece	[82]
11	158.00	Lamiaceae herb	*Hyssopus officinalis*	Greece	[82]
12	71.00	Lamiaceae flower	*Lavandula vera*	Greece	[82]
13	1018.00	Lamiaceae leaves	*Mentha pulegium*	Greece	[82]
14	1349.00	Araceae rhizome	*Acorus calamus*	Poland	[83]
15	7693.00	Asteraceae herbal	*Achillea millefolium*	Poland	[83]
16	7985.00	Asteraceae leaves	*Echinacea purpurea*	Poland	[83]
17	11,020.00	Lamiaceae herbal	*Rosmarinus officinalis*	Poland	[83]
18	2893.50	Papaveraceae herbal	*Chelidonium majus*	Poland	[83]
19	50.82	Raspberry herb	*Rubus* sp.	Australia	[84]
20	16.56	Thyme herb	*Thymus vulgaris*	Australia	[84]
21	6.76	Camomile herb	*Matricaria chamomilla*	Australia	[84]
22	2560.60	Oleaceae leaves	*Olea europaea*	Bulgaria	[85]
23	826.10	Oleaceae leaves	*Olea europaea*	Bulgaria	[85]
24	186.70	Vitaceae berries	*Vitis vinifera*	Czech Republic	[86]
25	1243.10	Vitaceae stem	*Vitis vinifera*	Czech Republic	[86]
26	659.40	Vitaceae leaves	*Vitis vinifera*	Czech Republic	[86]
27	50.12	Amaranthaceae leaves	*Achyranthes aspera*	India	[87]
28	196.39	Brassicaceae leaves	*Brassica juncea*	India	[88]
29	179.57	Brassicaceae seedlings	*Brassica juncea*	India	[88]
30	92.20	Tamaricaceace stem	*Tamarix aphylla*	Tunisia	[89]
31	668.00	Tamaricaceace leaves	*Tamarix aphylla*	Tunisia	[89]
32	594.45	Primulaceae leaves	*Labisia pumila*	Malaysia	[90]
33	1192.12	Primulaceae leaves	*Labisia pumila*	Malaysia	[90]
34	307.64	Asteraceae leaves	*Artemisia absinthium*	South Korea	[91]

**Table 2:** Synergic effect of honey with other natural products.

No.	Natural Products	Key Features	References
1	garlic raw extract	It has been shown that there is synergic antimicrobial activity between raw garlic extract and honey, but the antioxidant activity of the mixture is lower than that of *A. sativum*. Moreover, the mix of honey–*A. sativum extract* presented increased wound healing capacity compared to Euphorbia honey.	[98]
2	garlic	The mixture of garlic extracts and Tengen honey has significant sensitivity against Gram-positive and Gram-negative bacteria, which leads to their potential to treat infectious diseases.	[102]
3	rifampicin	Mixing Medihoney and rifampicin against laboratory and clinical strains of *S. aureus*, including MRSA strains, demonstrated a good synergism effect. Also, results support the potential of combining Manuka honey and antibiotics in treating *S. aureus*-related skin infections.	[105]
4	basil	The combined effect of honey and plants showed synergism. This fact is explained by the antibacterial effect that can be extremely useful in treating infested lesions and can also be used as a potential antimicrobial.	[115]
5	sweet basil	The synergic effects of honey combined with the alcoholic extract of *O. basilicum* appeared to have essential properties that made it useful as a wound dressing.	[120]
6	cinnamon	The study reveals that cinnamon could improve honey’s antibacterial effect against *S. mutans*.	[121]
7	ginger	*In vitro* data from this study shows antimicrobial activity has a synergic effect by combining ginger and honey against bacterial cells isolated from carious teeth. This investigation suggests that a paste made by blending ginger and honey could be used as a mouthwash in treating dental caries, mouth sores, and sore throats and could be incorporated into toothpaste to prevent dental caries.	[127]
8	ginger	Results confirmed that ginger strongly improves the MIC of honey, thus letting hope for a honey benefit and would constitute an alternative way against the resistance to bacteria.	[128]

**Table 3:** Honey mechanism of action.

No.	Type of Bee Product	Mode of Administration	Effects of Honey	References
1	honey (from the lime tree, chestnut, rapeseed, eucalyptus, and heather)	Oral administration of 1.2 g honey/kg body	↑ (high) β-carotene ↑ GSH (glutathione) ↑ vitamin C ↑ uric acid	[175]
2	honey	Oral administration of 1.2 g honey/kg body weight (7 men and 3 women) for 2 weeks	↑ serum vitamin C, iron, and copper levels ↑ β-carotene ↑ uric acid ↑ GSH ↑lymphocyte and eosinophil % ↓ (low) liver enzymes (AST—aspartate aminotransferase, ALT-alanine aminotransferase) ↓ serum Ig E (imunoglobulin E) ↓ lactic acid dehydrogenase ↓ creatinine kinase ↓ monocytes up to 50%	[176]
3	honey-pollen mix	*in vivo* induced liver injury through oral administration of acetaminophen on mice	↑ TBARS (thiobarbituric acid reactive substance) ↓ GSH in liver	[177]
4	honey	Oral administration of honey combined with metformin or glibenclamide on rats	↑ CAT (catalase), GR (glutathione reductase), TAS (total antioxidant status) and GSH for honey combined with metformin or glibenclamide	[178]
5	honey	0.2, 1.2 and 2.4 g honey/kg body weight/day -oral administration on rats	↑SOD (superoxide dismutase) ↓ TAS, CAT, GPx (glutathione peroxidase), GR and GST (glutathione S-transferase)	[179]
6	honey	Intravenous administration (2 g honey/kg body weight of sheep)	↑ NO (nitric oxide) levels	[176]
7	Tualang honey	Oral administration of 1.5 g/kg or 0.75 g honey/kg body weight on two groups of woman athletes	↑ ROS levels in athletes that consumed 1.5 g honey/ kg body	[180]
8	honey	Oral consumption: 1, 1.5 and 2 g/kg/day for every 2 weeks	↓ body weight, total cholesterol, low-density lipoprotein cholesterol, and triglyceride ↑ high-density lipoprotein-cholesterol, haemoglobin A_1c_	[181]

“↑”-high and “↓”-low quantity.
